# Clinically actionable molecular alterations in Rb-retained small cell lung carcinoma variants

**DOI:** 10.1007/s00428-026-04458-6

**Published:** 2026-03-11

**Authors:** Martin Zacharias, Nikolaus John, Karl Kashofer, Helmut Popper

**Affiliations:** 1 Diagnostic and Research Institute of Pathology, Medical University of Graz, Graz, Austria; 2 Division of Pulmonology, Medical University of Graz, Graz, Austria

**Keywords:** Pulmonary neuroendocrine neoplasms, Small cell lung carcinoma, RB1, Molecular profiling, Targeted therapy

## Abstract

Small cell lung carcinoma (SCLC) is classically defined by biallelic inactivation of *RB1* and *TP53*. However, a small subset of tumors retains Rb expression and exhibits distinct molecular features. Here, we report two Rb-retained SCLC cases that expand the biological and therapeutic spectrum of this subgroup. Both tumors occurred in middle-aged women, showed small cell morphology with some variant features, and displayed complex copy number alterations. Case 1 harbored a truncal *KRAS* p.G12C mutation with high-level amplification of chromosome 11q13-q14, including *CCND1*, and demonstrated a clinical response to sotorasib. Case 2 harbored a *TP53* mutation, *CDKN2A* loss, *STK11* inactivation, and a novel *IKZF2::ERBB4* fusion. These findings highlight the molecular heterogeneity of Rb-retained SCLC and demonstrate that this subgroup can harbor clinically actionable oncogenic drivers. Accordingly, routine assessment of Rb expression in SCLC, followed by comprehensive molecular profiling of Rb-retained tumors, is warranted to uncover therapeutically relevant targets.

## Introduction

Small cell lung carcinoma (SCLC) is traditionally characterized by biallelic inactivation of *RB1* and *TP53*, leading to loss of Rb protein expression and dysregulated cell-cycle control. However, integrated genomic and immunohistochemical analysis in a large cohort of SCLC cases has identified a minority (~ 6%) of tumors that retain Rb protein expression. These Rb-retained SCLC tumors display distinct biological features, including recurrent cyclin D1 overexpression/*CCND1* amplification, and enrichment for genetic alterations more commonly associated with non-small cell lung cancer (NSCLC) [1]. Within this biological spectrum, a subset of tumors lacking both *RB1* and *TP53* alterations has recently been designated “atypical SCLC”, characterized by extensive chromothripsis and molecular features linking them to pulmonary carcinoids [2]. Together, these observations highlight substantial heterogeneity and lineage plasticity within pulmonary neuroendocrine neoplasms [3]. Despite these insights, actionable oncogenic drivers in these rare SCLC variants remain poorly defined, limiting opportunities for precision therapy.

Here, we describe two Rb-retained SCLC cases with complex genomic profiles that further expand this emerging biological spectrum. In contrast to conventional SCLC, both tumors harbored potentially targetable molecular alterations, including a *KRAS* p.G12C mutation with documented response to the KRAS^G12C^ inhibitor sotorasib, and a novel *IKZF2::ERBB4* fusion. These cases underscore the clinical relevance of identifying Rb-retained SCLC variants and support routine Rb immunohistochemistry followed by comprehensive molecular profiling to uncover therapeutically actionable vulnerabilities.

## Materials and methods

### Histopathology and immunohistochemistry

Formalin-fixed, paraffin-embedded (FFPE) tumor tissue was evaluated using routine hematoxylin and eosin (H&E) staining. Routine tissue processing and immunohistochemistry (IHC) was performed according to accredited quality standards (EN ISO 15189) of the Diagnostic and Research Institute of Pathology, Medical University of Graz.

### Molecular profiling

Comprehensive genomic profiling was performed using the Oncomine Comprehensive Assay Plus (DNA- and RNA-based), detecting genomic alterations from 517 genes (single-nucleotide variants, insertions, deletions, copy number variations, and fusions) as well as genomic signatures such as tumor mutational burden (TMB) and microsatellite instability (MSI). Furthermore, low-density whole-genome sequencing was performed to evaluate larger copy number variations, as previously described [4]. All molecular analyses were performed according to manufacturers´ protocol recommendations and accredited quality standards (EN ISO 15189) of the Diagnostic and Research Institute of Pathology, Medical University of Graz.

## Results

### Case 1

**Fig. 1:**
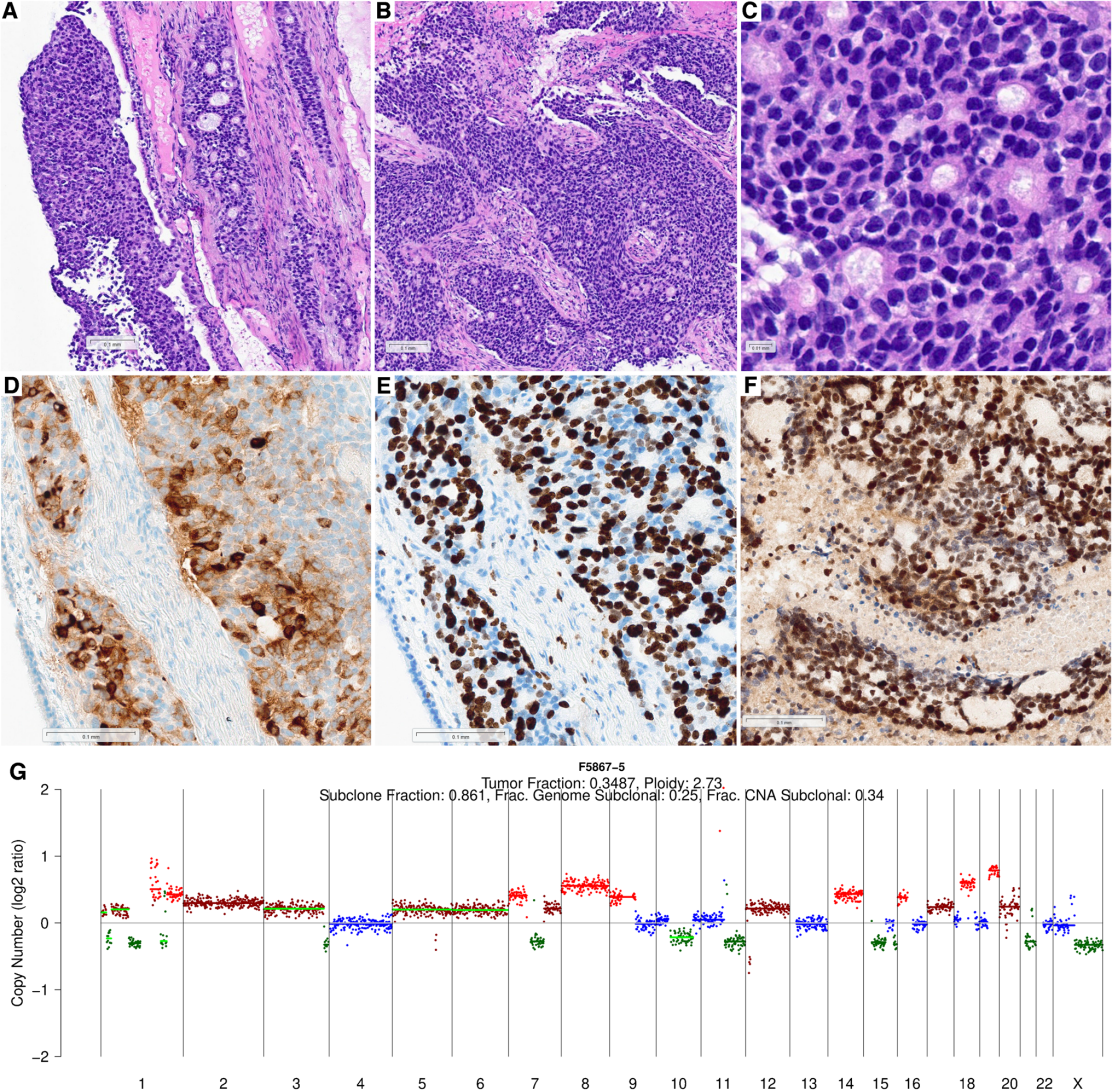
Histomorphology and immunohistochemical findings of case 1. Low-power views (**A**, **B**) show a subbronchially located tumor with partly solid and prominent rosette-like growth patterns. High-power examination highlights these rosette-like structures (**C**). Immunohistochemistry demonstrates synaptophysin positivity (**D**), a Ki67 proliferation index of approximately 70% (**E**), and retained nuclear Rb expression (**F**). Low-density whole-genome sequencing revealed a complex pattern of copy number variations (**G**)

A 54-year-old woman with a 60 pack-year smoking history presented with a 10.8 cm right lower lobe pulmonary mass causing bronchial compression and atrial infiltration. Subsequent radiological staging was suspicious for innumerable metastases within both lungs, lymph nodes, bone, and brain. Of note, neuron-specific enolase (NSE) concentration in peripheral blood was markedly elevated (120.9 ng/mL). Bronchoscopic lung and lymph node biopsies histologically confirmed epithelial tumor formations with partly solid, but often also prominent rosette-like growth patterns (Fig. 1). The tumor cells were small to intermediate-sized with scant cytoplasm, high nuclear-to-cytoplasmic ratio, dense evenly distributed chromatin, minor nuclear pleomorphism, and few atypical mitoses. All available biopsy material was thoroughly reviewed, and no morphologically or immunophenotypically distinct non-small cell component was identified. Immunohistochemically, tumor cells were positive for synaptophysin (approximately 50%), NeuroD1 (approximately 60%), pan-cytokeratin, and TTF-1, while negative for chromogranin, NCAM, POU2F3, and ASCL1. Napsin A demonstrated weak granular cytoplasmic staining, and lacked the strong, diffuse pattern typically seen in pulmonary adenocarcinoma. SMARCA4/BRG1 was retained. The Ki67 index was approximately 70%, and Rb expression was retained.

Molecular profiling of the tumor (estimated tumor cell content 40%) revealed a truncal *KRAS* p.G12C mutation (VAF 44.5%), and a pathogenic frameshift mutation in *MGA* (p.G1947Vfs*3, VAF 42.52%). Furthermore, two variants of uncertain significance were detected in *ATM* (p.Q2714E, VAF 71.61%) and *STAG2* (p.D385H, VAF 39.55%). Low-density whole-genome sequencing revealed copy number variations in nearly all chromosomes (Fig. 1G), with pronounced amplification of the chromosomal regions 11q13.2–13.3, 11q14.1, and 15q11.2, encompassing oncogenes such as *CCND1* (cyclin D1). Of note, cyclin D1 overexpression was also demonstrated on the protein level by IHC. Tumor mutational burden (TMB) was low (4.94 mut/Mb), microsatellite instability (MSI) testing was negative (score 1.46), and no gene fusions were detected. Nine months after diagnosis and subsequent failure of initial chemotherapy, the patient was then treated in second line with the KRAS^G12C^ inhibitor sotorasib, resulting in a clinically significant radiographic response that was ongoing at 4 months of follow-up.

### Case 2

**Fig. 2:**
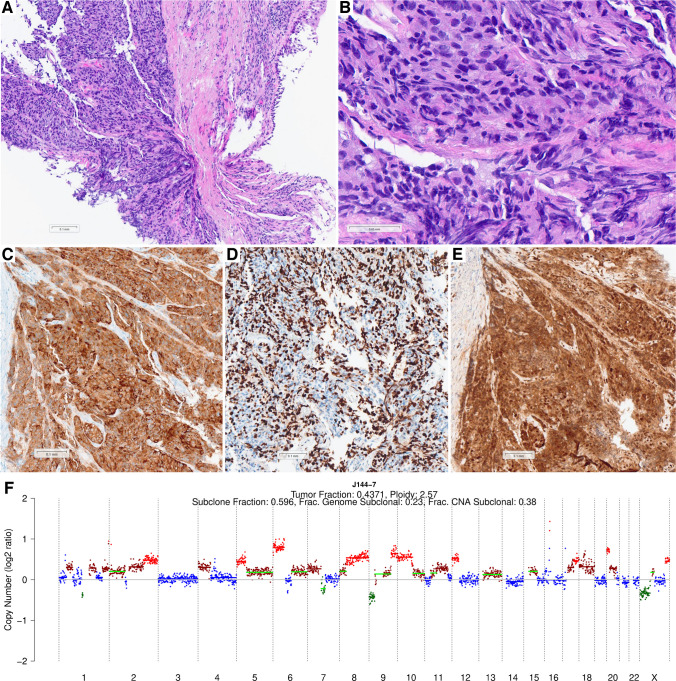
Histomorphology and immunohistochemical findings of case 2. Low-power view (**A**) shows a subbronchially located tumor with solid/diffuse growth pattern. High-power examination highlights small to intermediate-sized tumor cells with variable, often scant cytoplasm and occasional crush artifacts (**B**). Immunohistochemistry demonstrates synaptophysin positivity (**C**), a Ki67 proliferation index of approximately 80% (**D**), and retained nuclear Rb expression (**E**). Low-density whole-genome sequencing revealed a complex pattern of copy number variations (**F**)

A 55-year-old woman (previous smoker, pack-years unknown) presented with a 1.8 cm subbronchial pulmonary mass and intrathoracic lymphadenopathy suspicious for malignancy. Both the initial biopsy and the recurrence biopsy 1.5 years after diagnosis revealed epithelial tumor formations with comparable morphological and immunohistochemical features. The tumor histoarchitecture was solid/diffuse, the tumor cells were small to intermediate-sized with cytoplasm of varying width, often scarce (Fig. 2). All available biopsy material was thoroughly reviewed, and no morphologically or immunophenotypically distinct non-small cell component was identified. Immunohistochemically, tumor cells were positive for synaptophysin, NCAM (partially), INSM1 (partially), NeuroD1, pan-cytokeratin, and TTF-1, while negative for p40, ASCL1, POU2F3, Cyclin D1, and Napsin A. SMARCA4/BRG1 was retained. The Ki67 index was approximately 80%, and Rb expression was retained.

Molecular profiling of the tumor (estimated tumor cell content 50%) revealed pathogenic or likely pathogenic mutations in *SOS1* (p.N233Y, VAF 56.54%), *TP53* (p.R248L, VAF 46.32%), *STK11* (p.G242V, VAF 46.01%), and *BAP1* (p.E454Sfs*117, VAF 17.85%). Additional variants of uncertain significance were detected in *FLT4* (p.R642S, VAF 51.95%), *FAT1* (p.E2865G, VAF 50.18%), *ID3* (p.Q66L, VAF 38.83%), *ADAMTS12* (p.Y636*, VAF 28.35%), *ATRX* (p.G1964V, VAF 5.68%), and *KDM5C* (p.E914Q, VAF 5.03%). Importantly, RNA-based analysis identified an *IKZF2::ERBB4* fusion, involving *IKZF2* exon 4 fused to *ERBB4* exon 2, preserving the *ERBB4* coding region and consistent with a gain-of-function oncogenic event. The TMB was modestly elevated (11.43 mut/Mb), and MSI testing indicated microsatellite stability (score 0). Low-density whole-genome analysis revealed copy number variations on nearly all chromosomes (Fig. 2F), with pronounced chromosomal gains at 6p, 16p11.1–12.1, 20p12.2–13, and Xq26.1–28, as well as loss at 9p21.2–24.3, encompassing *CDKN2A* (the latter confirmed in the OCA + assay). The patient was treated with several lines of chemoradiotherapy and died 22 months after initial diagnosis. Clinicopathological and molecular findings of both cases are summarized in Table 1.

**Table 1:** Clinicopathological and molecular features of Rb-retained SCLC variant cases

Feature	Case 1	Case 2
Age/sex	54/female	55/female
Smoking history	Former smoker (60 py)	Former smoker (py unknown)
Primary site	Lung, right lower lobe	Lung, left upper lobe
Location within lung lobe	Central	Central
Neuron-specific enolase (NSE)	NSE elevated (120.9 ng/mL)	Not available
Histomorphology	Small cell, rosette-like	Small cell, solid/diffuse
Neuroendocrine IHC markers	Synaptophysin +, NeuroD1 +	Synaptophysin +, NCAM +, INSM1 +, NeuroD1 +
Ki67 IHC (%)	70	80
Cytokeratin/TTF1 IHC	PanCK +, TTF1 +	PanCK +, TTF1 +
*RB1* status (IHC/genomic)	Retained	Retained
*TP53* status	Wild-type	Mutated (LOF)
Further mutated genes*	*KRAS* (GOF), *MGA* (LOF)	*STK11* (LOF), *SOS1* (GOF), *BAP1* (LOF)
TMB	4.94 mut/Mb	11.43 mut/Mb
MSI	MSS (score 1.46)	MSS (score 0)
Gene fusions	None	*IKZF2::ERBB4*
CNV burden	High	High
Actionable alteration	*KRAS* p.G12C	*ERBB4* fusion
Targeted therapy received	Sotorasib (partial response)	None

*Pathogenic or likely pathogenic sequence variants only

Abbreviations:* py*, pack-years;* IHC*, immunohistochemistry;* GOF*, gain-of-function;* LOF*, loss-of-function;* TMB*, tumor mutational burden;* MSI*, microsatellite instability;* MSS*, microsatellite stable; *CNV*, copy number variation

## Discussion

Our case series further expands the emerging spectrum of Rb-retained SCLC and provides additional evidence that this biologically distinct subgroup may harbor therapeutically relevant genomic alterations. Consistent with prior observations [1, 2], both tumors in our series retained Rb expression, showed high proliferative indices, and exhibited extensive chromosomal restructuring. Importantly, both cases harbored potentially actionable oncogenic alterations, underscoring the clinical relevance of recognizing Rb-retained SCLC and pursuing extended molecular profiling in such cases.

The first case demonstrates that oncogenic *KRAS* activation can act as a dominant driver in Rb-retained SCLC variants. Although the tumor showed some remarkable variant features, the integrated morphological, immunohistochemical, clinical, and molecular findings support classification as Rb-retained SCLC rather than adenocarcinoma or combined SCLC/NSCLC. Importantly, it has been shown that weak granular Napsin A expression (as in our case) does not exclude high-grade pulmonary neuroendocrine carcinoma [5]. In contrast to NSCLC, *KRAS* mutations are exceedingly rare in classical SCLC [6, 7]. To our knowledge, we describe the first report of an Rb-retained SCLC harboring a *KRAS* p.G12C mutation with documented clinical response to sotorasib, a novel therapeutic molecule selectively and irreversibly targeting KRAS^G12C^ across solid tumors [8, 9]. Thus, we highlight a clear therapeutic opportunity in this molecular subset.

The second case further illustrates the molecular diversity of Rb-retained SCLC, with co-occurring *TP53* mutation, *CDKN2A* loss, *STK11* inactivation, and a novel *IKZF2::ERBB4* fusion. This constellation suggests convergence between neuroendocrine lineage programs and oncogenic signaling pathways typically associated with NSCLC. *ERBB4* rearrangements have been reported as recurrent oncogenic events in several solid tumors [10, 11], yet have not previously been described in SCLC, making this observation particularly noteworthy. In addition, the presence of an inactivating *STK11* mutation is of interest, as recent large-scale analyses have identified a small (1.7%) but clinically relevant subset of *STK11*-mutant SCLC associated with adverse prognosis [12], in line with the dismal course of our patient. Although rare in their overall cohort, *STK11*-mutant tumors harbored fewer mutations in *RB1* [12], potentially pointing towards an enrichment in Rb-retained SCLC. Given that *STK11* mutations in NSCLC are associated with decreased response to immune checkpoint inhibitors [13], molecular testing in Rb-retained SCLC might also lead to better stratification of patients for immunotherapy.

Taken together, our findings support emerging models of lineage plasticity in pulmonary neuroendocrine neoplasms, in which Rb-retained SCLC may arise through alternative evolutionary trajectories distinct from conventional SCLC. Within this framework, chromosomal instability and focal oncogene amplification, rather than Rb loss, may serve as primary oncogenic drivers. This concept aligns with recent molecular studies suggesting overlap between carcinoid tumors, high-grade neuroendocrine carcinomas, and transdifferentiated epithelial malignancies [1–3, 14]. Specifically, the presence of canonical NSCLC-associated drivers such as *KRAS* (case 1) and *STK11* (case 2) supports the concept that a subset of Rb-retained SCLCs originate from non-neuroendocrine progenitors (potentially alveolar type II or basal cells) that have undergone a complete phenotypic shift towards a neuroendocrine lineage [3]. This phenotypic plasticity may also account for the presence of variant (“adenocarcinoma-like”) features observed in our cases. Alternatively, the presence of an unsampled non-small cell component cannot be entirely excluded; however, all available biopsy material was thoroughly reviewed, and no morphologically or immunophenotypically distinct non-small cell component was identified.

From a diagnostic standpoint, our findings support incorporating Rb immunohistochemistry as a routine component of SCLC evaluation. Identification of Rb-retained cases should prompt comprehensive molecular profiling, as these tumors may harbor clinically actionable alterations not typically expected in conventional SCLC. Recognition of this subgroup has thus direct therapeutic implications and supports a more nuanced, biology-driven classification of pulmonary neuroendocrine neoplasms.
